# Large‐Scale MXene Membrane Fabrication via Nonsolvent Phase Separation

**DOI:** 10.1002/smtd.202502288

**Published:** 2026-05-01

**Authors:** Mostafa Dadashi Firouzjaei, Zahra Zandi, Hesam Jafarian, Anupma Thakur, Sanam Etemadi Maleki, Ahmad Rahimpour, Ahmad Arabi Shamsabadi, Mohtada Sadrzadeh, Babak Anasori, Mark Elliott

**Affiliations:** ^1^ Department of Civil Construction and Environmental Engineering University of Alabama Tuscaloosa Alabama USA; ^2^ Department of Mechanical Engineering 10–241 Donadeo Innovation Center for Engineering Advanced Water Research Lab (AWRL) University of Alberta Edmonton AB Canada; ^3^ School of Materials Engineering Purdue University West Lafayette Indiana USA; ^4^ Department of Chemistry University of Pennsylvania Philadelphia Pennsylvania USA; ^5^ School of Mechanical Engineering Purdue University West Lafayette Indiana USA

**Keywords:** industrial‐scale fabrication, mixed‐matrix membranes, nonsolvent induced phase separation, roll‐to‐roll processing, Ti3C2Tx‐polysulfone membranes, water purification

## Abstract

Over the past decade, 2D MXenes have garnered tremendous attention for membrane applications owing to their exceptional hydrophilicity, tunable surface chemistry, and antifouling properties. However, translating MXene‐based membranes from laboratory demonstrations to practically relevant manufacturing remains challenging because scalable fabrication routes are still limited. Herein, we demonstrate a roll‐to‐roll nonsolvent‐induced phase separation (R2R‐NIPS) process for fabricating Ti_3_C_2_T*_x_*/polysulfone (PSF) mixed‐matrix ultrafiltration membranes in large sheet format (196 × 84 cm; geometric area ≈1.65 m^2^). Incorporation of 1 wt% MXene produced more open and interconnected membrane substructures, enhanced apparent wettability (contact angle reduced from 90° for PSF to 62°), a more negative surface charge, and subsurface flake embedding confirmed by characterization. These features yielded a water flux of 206 LMH (31% higher than pristine PSF) with 97.6% humic acid rejection (*n* = 3 batches, *p* < 0.01 for flux; *p* < 0.05 for rejection), while batch‐to‐batch variability remained <5%. Rather than claiming a measured thermodynamic phase diagram, we interpret the observed morphology as consistent with faster demixing during R2R‐NIPS. This processing methodology—documenting practical parameter choices such as casting speed, wet thickness, and bath circulation—provides a feasible route toward larger‐scale MXene membrane production for water purification.

## Graphical abstract

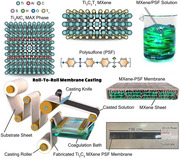

## 1 Introduction

Over the past decade, MXenes, particularly Ti_3_C_2_T*_x_*, have garnered tremendous attention due to their special properties, including rich surface chemistry, hydrophilicity, negative surface charge, and versatility [1, 2]. This has positioned MXenes as promising materials for a wide range of applications, from energy storage to environmental remediation [2, 3, 4, 5]. However, despite these advances, the translation of MXene‐based application‐driven products from laboratory‐scale studies to industrially relevant manufacturing remains challenging [4, 5, 6]. Therefore, addressing the scalability of MXene‐based products is essential to unlocking their full potential and enabling their integration into real‐world technologies.

Efforts for the mass production of MXenes have been gaining momentum in recent years [7, 8, 9]. A review of the current MXene synthesis methods highlights advances in both top‐down and bottom‐up techniques to produce MXenes with high purity and reproducibility on a larger scale [7, 8, 9]. For instance, fluoride‐free etching methods have been explored to improve production efficiency while reducing environmental impact [8, 9]. One example is the study by Wang et al., which introduces a rapid, scalable, low‐temperature molten salt etching method for Ti_3_C_2_T*_x_* synthesis, enabling large‐scale production within minutes [9]. Additionally, researchers have started integrating MXene materials into multifunctional structures such as films, fibers, and membranes to expand their industrial applicability [10, 11].

Our previous work has emphasized the importance of understanding the environmental impact of Ti_3_C_2_T*_x_* MXene production [6]. Through a comprehensive life cycle analysis, we identified key challenges and opportunities in scaling up MXene synthesis while minimizing ecological impacts [6]. Building on this foundation, we now explore the feasibility of a large‐scale nonsolvent phase separation (NIPS) technique for fabricating MXene‐based membranes.

Roll‐to‐roll NIPS (R2R‐NIPS) membrane fabrication could be the primary approach for large‐scale manufacturing of MXene‐based membranes due to its high efficiency, scalability, and cost‐effectiveness [12, 13]. This continuous processing technique allows for the rapid production of membranes over large areas, overcoming the limitations of batch‐based casting methods that are slow and inefficient for industrial applications [12, 13]. Given the inherent hydrophilicity and special structure of Ti_3_C_2_T*_x_*, R2R‐NIPS fabrication enables precise control over coating thickness, ensuring uniform distribution of Ti_3_C_2_T*_x_* MXene flakes within the polymer matrix while preventing aggregation [10, 11, 12, 13]. Additionally, the integration of R2R‐NIPS processing provides a controlled environment that optimizes pore formation and enhances membrane performance [12, 13]. This method also supports real‐time monitoring and automation, ensuring consistency in membrane quality and reducing material waste.

Despite these clear advantages, large‐area MXene coatings and films have only recently begun to emerge for specific formats such as slot‐die‐coated nanofiltration layers [10, 11], whereas large‐scale R2R‐NIPS fabrication of Ti_3_C_2_T*_x_*/polymer mixed‐matrix ultrafiltration membranes remains scarcely reported. One key barrier has been the intrinsic tendency of MXene flakes to oxidize or restack when processed under aqueous or poorly controlled conditions, leading to loss of their characteristic 2D morphology and transport advantages [14, 15]. Another significant challenge has been formulating a MXene dispersion that remains stable and sufficiently flowable for continuous coating [15, 16]. In addition, integration of MXene inks into existing R2R‐NIPS lines requires tailored solvent and coagulation conditions to prevent premature flake aggregation or membrane delamination—challenges that have discouraged adaptation of conventional polymeric membrane machinery to MXene‐containing systems. By addressing these processability and material‐stability hurdles, our work advances the translation of MXene nanosheet science from benchtop membranes toward continuous manufacture.

In this study, we present large‐scale MXene‐based membrane fabrication through the R2R‐NIPS process and demonstrate the scalability of MXene‐based polysulfone (PSF) ultrafiltration membranes for water purification applications. We also discuss the practical processing parameters governing continuous fabrication and provide a conservative, morphology‐based interpretation of membrane formation during NIPS. This work highlights the potential of Ti_3_C_2_T*_x_*‐PSF membranes in water treatment and their broader relevance to industries that require advanced material solutions.

## 2 Experimental

### 2.1 Synthesis of Ti_3_C_2_T*_x_* MXene

**FIGURE 1:**
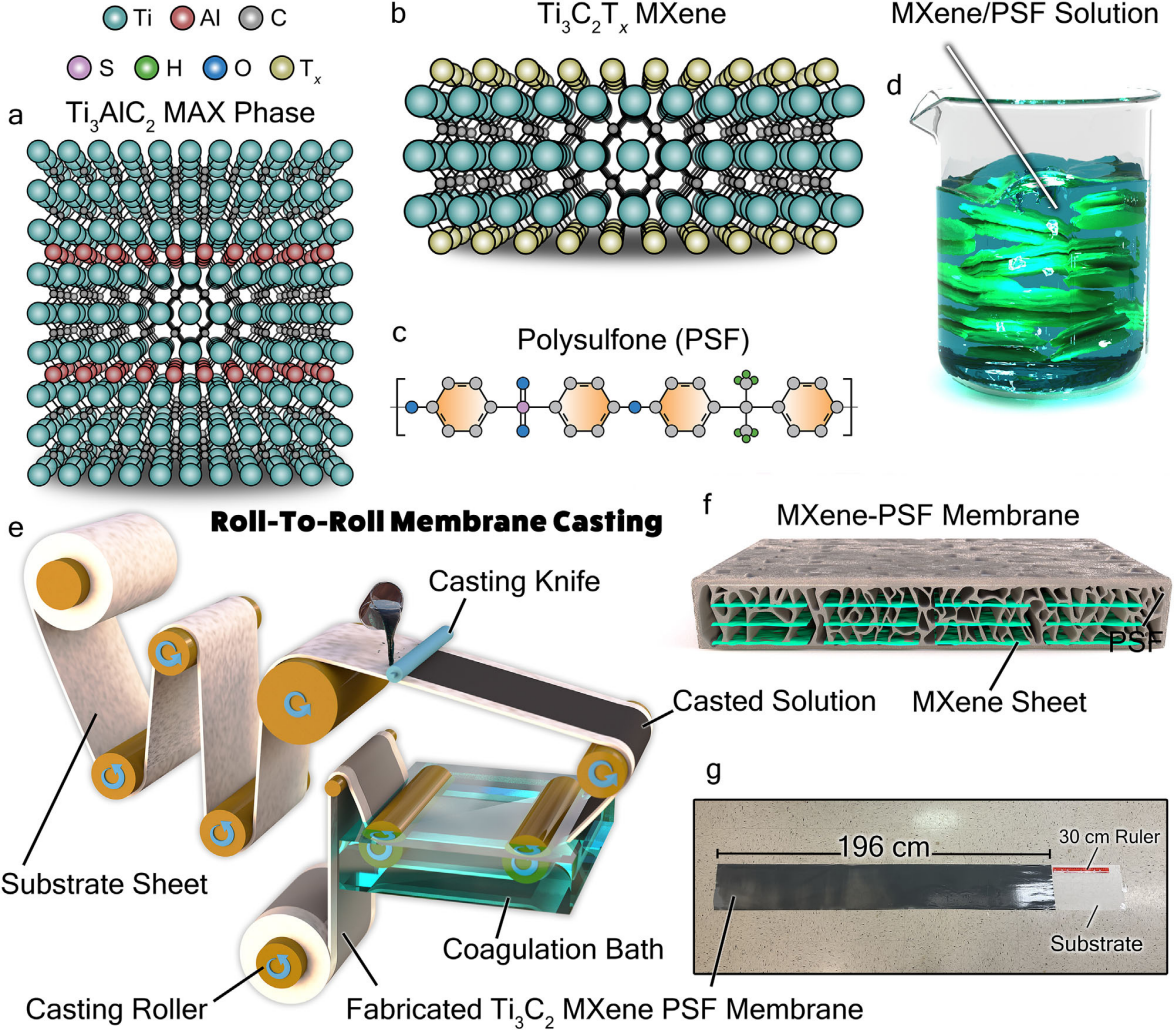
(a, b) Schematic illustration of Ti_3_AlC_2_ MAX and Ti_3_C_2_T*_x_* MXene chemical structure, (c) chemical structure of polysulfone (PSF), (d) schematic of a MXene‐PSF solution, (e) schematic of the roll‐to‐roll (R2R) casting machine used for membrane fabrication, (f) schematic of a MXene‐PSF mixed matrix membrane, (g) a digital photograph of the 196×84 cm Ti_3_C_2_ ‐PSF membrane taped on the laboratory floor.

The Ti_3_AlC_2_ MAX phase was synthesized by combining titanium carbide, titanium, and aluminum powders in a molar ratio of 2:1:1.1 and thoroughly mixed using a jar mill for 18 h [17]. The resultant precursor powder was packed into an alumina crucible and placed in a high‐temperature tube furnace (Carbolite Gero, 1700°C model) for pressureless sintering at 1400°C for 4 h under a constant argon flow. After sintering, the sintered block of Ti_3_AlC_2_ was ground into a fine powder using a TiN‐coated mill bit, and sieved through a 70 µm mesh. Figure 1a shows the schematic structure of the Ti_3_AlC_2_.

Ti_3_C_2_T*_x_* MXene was synthesized using a mixed‐acid wet‐chemical etching approach. Briefly, for 1 g of the synthesized Ti_3_AlC_2_ MAX phase, an etchant solution composed of a (6:3:1) mixture by volume of 12 M HCl, deionized water, and 28.4 M hydrofluoric acid (HF) was used. This mixture was stirred at 400 RPM for 24 h at 35°C to selectively etch the aluminum layers from the Ti_3_AlC_2_ MAX phase. Next, the etched product was washed with deionized water via repeated centrifugation (4–5 cycles with approximately 200 mL of water) at 3234 RCF until the supernatant reached a neutral pH (≈6). For delamination, the multilayered Ti_3_C_2_T*_x_* sediment was dispersed in a lithium chloride (LiCl, Sigma‐Aldrich) solution (50 mL per gram of etched powder). The mixture was stirred at 400 RPM for 1 h at 65°C under constant argon flow. Post‐reaction, the mixture was washed with deionized water by centrifugation at 3234 RCF for varying durations (5, 10, 15, and 20 min). The final suspension was vortexed for 30 min and centrifuged at 2380 RCF for 30 min to ensure the flakes were primarily single‐ to few‐layered. The resulting Ti_3_C_2_T*_x_* MXene suspension was stored at −20°C until use. Figure 1b illustrates the schematic structure of the Ti_3_C_2_T*_x_* MXene.

### 2.2 Fabrication of Large‐Scale MXene Membranes

Large‐scale membranes were fabricated using a roll‐to‐roll casting machine that was custom‐designed and built in‐house. Figure 1e shows the schematic of the casting machine constructed by our group for this work. Two types of membranes were fabricated: (a) a PSF membrane without MXene and (b) a Ti_3_C_2_‐PSF composite membrane with MXene. The compositions of the polymer solutions used for the membranes are presented in Table 1. Figure 1c presents the illustration of the PSF chemical structure, and Figure 1d illustrates the schematic of the MXene‐PSF solution.

**TABLE 1:** Fabricated membrane compositions and labeling.

Membrane∖ Compositions (wt.%)	Ti_3_C_2_T*_x_*	PSF	PVP	DMF
PSF	—	16.0	1.0	83.0
Ti_3_C_2_‐PSF	1.0	16.0	1.0	82.0

The polymer solutions were prepared by dissolving the PSF (Sigma‐Aldrich) in dimethylformamide (DMF, Sigma‐Aldrich) with continuous stirring for 6 h to ensure homogeneity. The resulting solutions were then degassed in a vacuum oven for 10 min to remove trapped air and prevent defects during membrane casting. The R2R casting machine was used to fabricate the membranes through a phase separation process. Polyester was used as the substrate for membrane formation. The polymer solution was cast onto the polyester substrate using the casting machine, with the thickness controlled by the machine's adjustable casting head, and the cast polymer films were immediately submerged in a deionized water coagulation bath. Unless otherwise noted, membranes were fabricated using a wet casting thickness of 200 µm, a line speed of 2.5 m min^−^^1^, a DI‐water coagulation bath at 21°C, and bath circulation of 2.0 L min^−^^1^. Using this setup, we fabricated a 196 × 84 cm Ti_3_C_2_‐PSF membrane (geometric area ≈1.65 m^2^). The fabrication of PSF membranes without MXene followed an identical protocol, ensuring consistency between the two types.

### 2.3 Membrane Characterization and Filtration

The performance of the fabricated membranes was evaluated through humic acid (HA) removal experiments using a cross‐flow filtration system. The feed solution consisted of humic acid at a concentration of 2000 ppm in deionized (DI) water. Prior to filtration, the membranes were compacted at 40 psi for 3 h using DI water to ensure stability and eliminate any initial flux variations. The coagulation bath temperature during membrane fabrication was 21°C, whereas all filtration experiments were conducted separately at a constant temperature of 25°C. The same cross‐flow filtration unit was used for all membrane testing. Humic acid removal was evaluated by collecting both the feed and permeate samples. The humic acid concentration in these samples was measured using a UV–Vis spectrophotometer at 254 nm. Statistical comparisons between membrane types were assessed using Student's t‐test on *n* = 3 independent R2R batches.

(1) $$R\left(\%\right)=\left(1-\frac{C_{p}}{C_{f}}\right)\times 100$$

where *R* is the rejection percentage, *C*_p_ is the humic acid concentration in the permeate, and *C*_f_ is the humic acid concentration in the feed. The water flux of the membranes was determined by measuring the mass of the permeate collected over time using a balance. The water flux (*J*) was calculated using Equation 2 below:

(2) $$J=\frac{V}{A\times t}$$

where *J* is the water flux (L m^−2^ h^−1^), *V* is the volume of the permeate collected (L), *A* is the effective membrane area (m^2^), and *t* is the filtration time. The results from the humic acid rejection experiments and water flux measurements were used to evaluate and compare the performance of the PSF and Ti_3_C_2_‐PSF membranes.

The synthesized Ti_3_C_2_T*_x_* MXenes and fabricated MXene‐PSF membranes were characterized to evaluate their structural, morphological, and surface properties. The Ti_3_C_2_T*_x_* MXenes were characterized using X‐ray Diffraction (XRD) and scanning electron microscopy (SEM). XRD analysis confirmed the crystalline phase and structural integrity of the synthesized Ti_3_C_2_T*_x_* MXene. SEM imaging was used to analyze the morphology and layered structure of the Ti_3_C_2_T*_x_* MXene flakes.

The fabricated membranes (both PSF and Ti_3_C_2_‐PSF membranes) underwent comprehensive characterization using a range of analytical techniques. SEM and transmission electron microscopy (TEM) were used to examine the surface and cross‐sectional morphologies as well as the distribution of MXene flakes within the membrane matrix. Atomic force microscopy (AFM) was employed to measure the surface roughness of the membranes. X‐ray photoelectron spectroscopy (XPS) provided insights into the chemical states of the membrane surfaces. At the same time, energy dispersive spectroscopy (EDX) was used for elemental mapping to confirm the presence and distribution of MXenes in the composite membranes.

Fourier transform infrared spectroscopy (FTIR) was conducted to identify the functional groups in the membranes and to verify chemical interactions between the MXene and polymer matrix. XRD was used to assess the crystalline structure of the membranes and to detect any changes induced by the addition of MXenes. Contact angle measurements were performed to determine the hydrophilicity of the membrane surfaces. Zeta potential analysis was carried out to evaluate the surface charge of the membranes, which is critical for understanding their antifouling and filtration performance.

## 3 Results and Discussion

### 3.1 Process Variables and Their Effects in R2R‐NIPS MXene Membrane Fabrication

**FIGURE 2:**
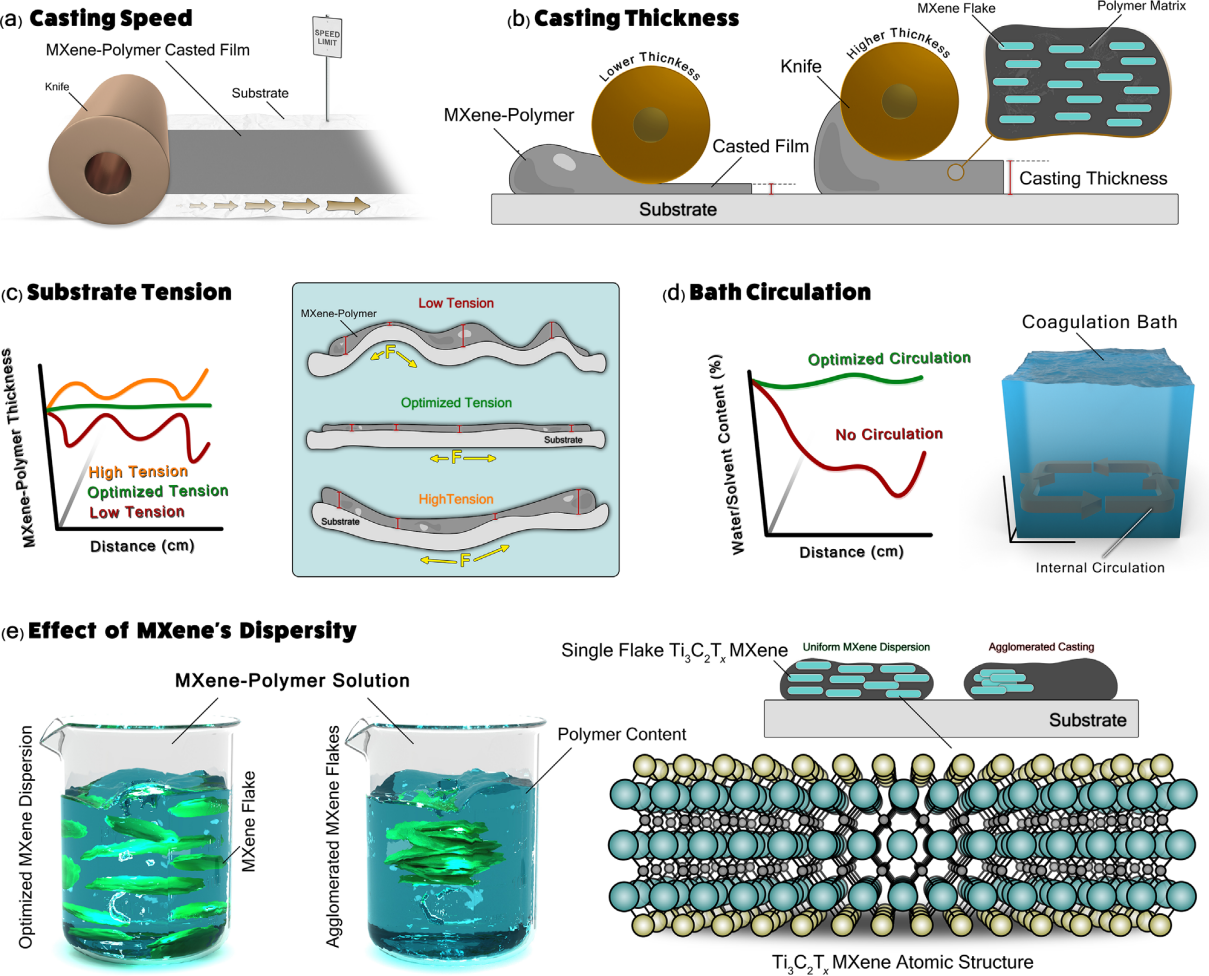
Conceptual schematic summarizing key process sensitivities in the R2R‐NIPS casting of Ti_3_C_2_T*_x_*‐PSF membranes. The illustrations are qualitative and are intended to show how casting speed, wet thickness, substrate tension, bath circulation, and dope dispersion can affect coating stability, defect formation, and membrane uniformity; they are not a quantitative optimization map.

Large‐scale Ti_3_C_2_T*_x_*/PSF membranes were fabricated using a R2R‐NIPS line, and the principal process sensitivities encountered during fabrication are summarized schematically in Figure 2. Here, Figure 2 should be read as a conceptual process map rather than a quantitative optimization chart. In practical terms, casting speed, wet thickness, substrate tension, bath circulation, and dope dispersion quality each affected coat uniformity, defect formation, and membrane recovery. The processing conditions used in this study (2.5 m min^−^^1^ line speed, 200 µm wet thickness, DI‐water bath at 21°C, and 2.0 L min^−^^1^ bath circulation) were selected on the basis of processability, stable continuous casting, and reproducible defect‐minimized membrane formation during iterative trials. Additional fabrication details are provided in the Supporting Information.

### 3.2 MXene and Membrane Characterization

**FIGURE 3:**
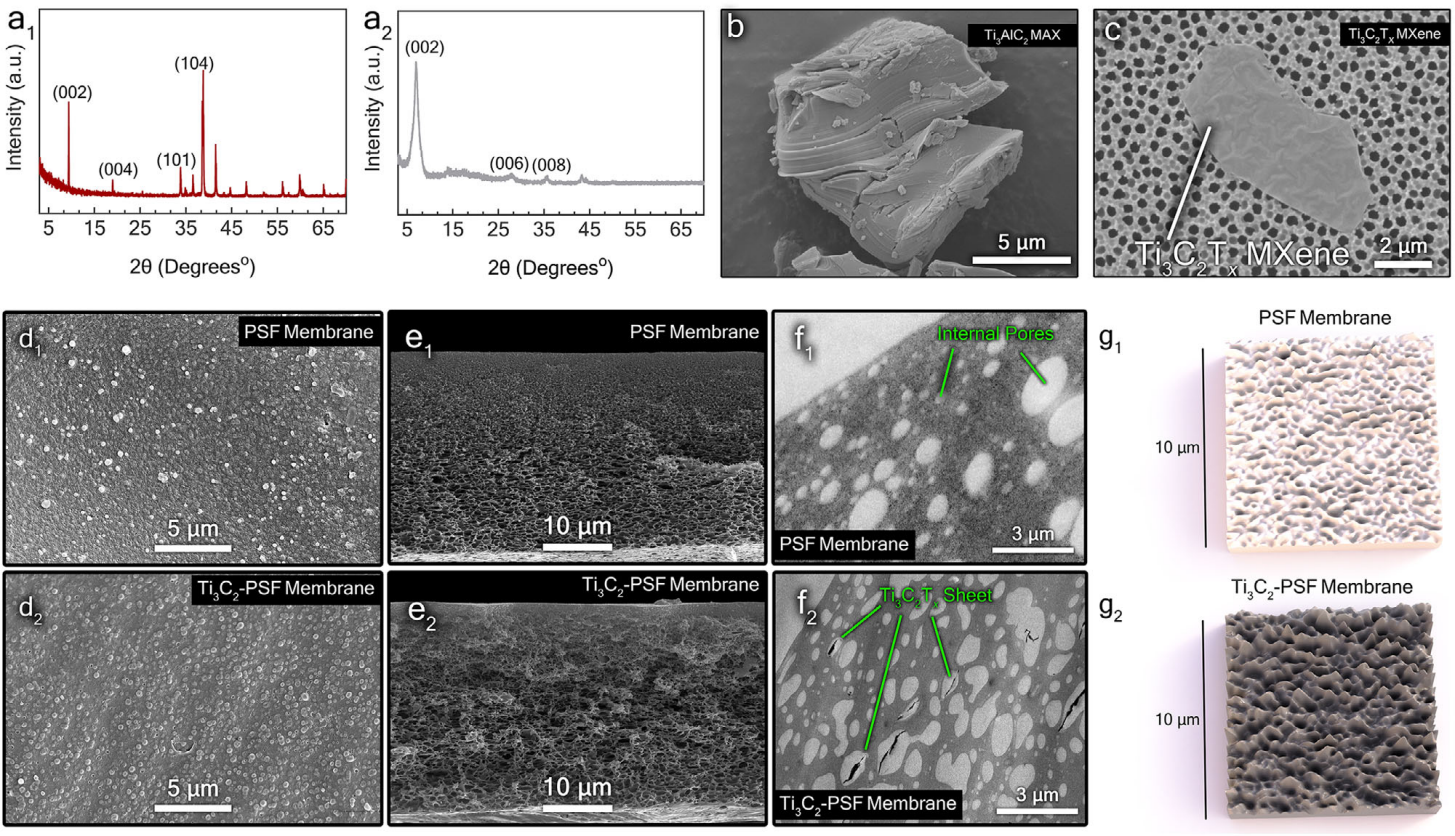
Characterization of Ti_3_C_2_T*_x_* and Ti_3_AlC_2_ MAX phase. XRD spectra of  (a_1_) Ti_3_AlC_2_ and (a_2_) Ti_3_C_2_T*_x_* MXene. (b) SEM image of Ti_3_AlC_2_, showing its bulky and layered structure. (c) SEM images of exfoliated Ti_3_C_2_T*_x _* flakes, highlighting their 2D sheet‐like morphology. (d_1_, d_2_) Top surface SEM images of PSF and Ti_3_C_2_‐PSF membranes, respectively, illustrating the altered morphology due to MXene addition. (e_1_, e_2_) Cross‐sectional SEM images of PSF and Ti_3_C_2_‐PSF membranes, showing the enhanced porosity and interconnected pore structure in the Ti_3_C_2_‐PSF membrane. (f_1_, f_2_) High‐resolution cross‐sectional TEM images of PSF (f1) and Ti_3_C_2_‐PSF (f2) membranes, directly visualizing dark, layered MXene flakes embedded 10–500 nm subsurface throughout the porous matrix, with none at the outermost polymer skin (scale bar 200 nm; inset f2: higher‐magnification flake encapsulation/orientation). This confirms subsurface distribution reconciling uniform AFM roughness and XPS Ti absence, per rapid NIPS skin formation excluding >5 nm particles. AFM images of (g_1_) the PSF membrane and (g_2_) the Ti_3_C_2_‐PSF membrane.

The structural and morphological properties of the Ti_3_C_2_T*_x_* and the fabricated membranes were analyzed to confirm the successful integration of MXene flakes into the mixed matrix membrane and to assess the impact of MXene addition on membrane characteristics. The XRD patterns in Figure 3a_1_ clearly show the characteristic peaks for Ti_3_AlC_2_ MAX phase confirming the synthesis of the Ti_3_AlC_2_ MAX. After the selective etching process, the (002) peak of Ti_3_AlC_2_ MAX at 9.42° shifted to a lower angle ≈6.91° as shown in Figure 3a_2_, suggesting the synthesis of Ti_3_C_2_T*_x_* MXene. Figure 3b presents SEM image of the synthesized Ti_3_AlC_2_ MAX phase with its characteristic bulk layered structure while Figure 3c displays the SEM image of the delaminated single‐to‐few layer Ti_3_C_2_T*_x_* MXene flake with lateral sizes of ≈3 µm.

Moving to the polymeric membranes, Figure 3d_1_,d_2_ shows the SEM image of the top surface of the membrane, which exhibits a typical smooth polymeric structure. Figure 3e_1_,e_2_ displays a cross‐sectional SEM image and the porous structure of the membranes. The addition of MXene results in larger pores with more interconnected channels, reflecting the impact of MXene on the phase inversion process and pore formation during membrane fabrication. Figure 3f_1_ shows the cross‐sectional TEM images of the membranes. Figure 3f_2_ reveals the presence of Ti_3_C_2_T*_x_* flakes within the pores and polymer matrix of the Ti_3_C_2_‐PSF membrane and also confirms that the addition of MXene increases the porosity of the membrane, with larger and more heterogeneous pore structures compared to the PSF membrane. These TEM micrographs capture the depth profile across multiple micrometers, showing MXene distributions predominantly in the sub‐surface regions (10–500 nm depth). This indicates that the inclusion of MXene significantly influences the phase inversion process, altering the internal membrane structure. The surface properties of the membranes were further examined using AFM, as shown in Figure 3g_1_,g_2_. AFM measurements of the membrane surfaces gave nearly identical arithmetic average roughness (*R*_a_) values: 12.23 ± 2.49 nm for the Ti_3_C_2_‐PSF composite versus 12.54 ± 1.21 nm for the neat PSF membrane. Because AFM scans only the outermost few nanometers of a surface, any MXene sheets lying exposed or protruding would register as additional peaks or valleys, increasing the measured roughness. The fact that the *R*_a_ values overlap within their measurement uncertainties means there were no detectable protrusions attributable to MXene flakes. From this, we infer that the MXene nanosheets must be buried just beneath the polymer‐rich top layer rather than sitting at the very surface. In other words, during the NIPS process, the polymer matrix likely flowed over and encapsulated the Ti_3_C_2_T*_x_* flakes, so that the outermost morphology remains governed by the PSF phase alone. This embedding preserves a smooth surface while still allowing subsurface MXene functionality.

**FIGURE 4:**
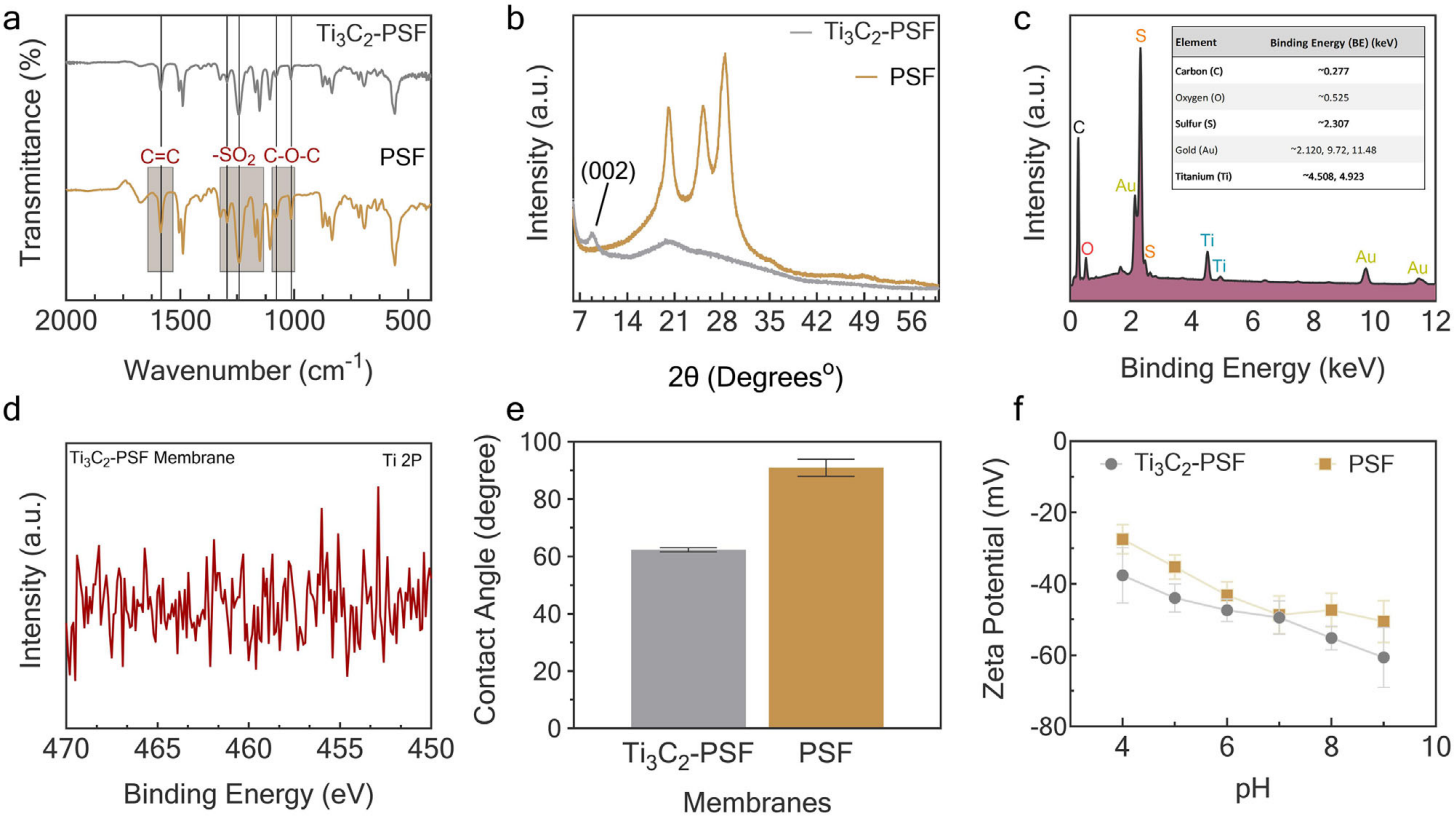
Characterization of Ti_3_C_2_‐PSF and PSF membranes: (a) FTIR spectra showing the chemical functional groups of the membranes, with distinct peaks corresponding to characteristic bonds in each membrane type. (b) XRD patterns confirming the structural differences between Ti_3_C_2_‐PSF and PSF membranes, including the (002) peak for Ti_3_C_2_T*_x_*. (c) EDX spectrum illustrating the elemental composition of the Ti_3_C_2_‐PSF membrane, with binding energy peaks corresponding to carbon (C), oxygen (O), sulfur (S), gold (Au), and titanium (Ti). (d) XPS spectrum for the Ti range peak in the Ti_3_C_2_‐PSF membrane, showing no detectable titanium signal, indicating that Ti_3_C_2_ MXene is embedded within the membrane rather than on the surface. (e) Contact angle measurements demonstrating the enhanced hydrophilicity of the Ti_3_C_2_‐PSF membrane compared to the PSF membrane. (f) Zeta potential analysis across a pH range, showing the increased negative surface charge of the Ti_3_C_2_‐PSF membrane due to the incorporation of negatively charged MXene flakes.

Figure 4a shows the FTIR spectra of the PSF membrane and the Ti_3_C_2_‐PSF membranes. The PSF membrane exhibits characteristic absorption bands typical of PSF, including stretching vibrations of sulfone (─SO_2_─) groups at ≈1150–1300 cm^−^^1^ [18], aromatic C═C stretching vibrations at ≈1500–1600 cm^−^^1^ [19], and aromatic ether (C─O─C) vibrations at ≈1000–1100 cm^−^^1^ [20]. The FTIR spectrum of the Ti_3_C_2_‐PSF membrane retains these characteristic PSF peaks but shows a slight reduction in the intensity of some sulfone and aromatic peaks, suggesting potential physical interactions between the MXene surface and the PSF functional groups. Importantly, the absence of significant new peaks indicates that the incorporation of Ti_3_C_2_ MXene into PSF primarily alters the physical interaction environment within the membrane without forming entirely new chemical bonds.

The PSF membrane XRD pattern displays a broad, amorphous halo typical of non‐crystalline polymers like polysulfone located at 2*θ* ≈ 20.1°, 25.0°, and 28.4°, indicating its lack of long‐range order (Figure 4b) [21]. In contrast, the XRD pattern of the Ti_3_C_2_‐PSF membrane retains the amorphous nature of the polymer 2*θ* ≈ 20.1° but also shows the characteristic peak of Ti_3_C_2_T_x_ at 2*θ* ≈ 8.7°, which is the (002) peak of Ti_3_C_2_T*_x_*, confirming the incorporation of MXene flakes into the polymer matrix. The reduced intensity and broadening of this peak suggest that the arrangement of the 2D flakes is disrupted due to their dispersion within the amorphous PSF matrix. The overall reduction in peak intensity and the absence of sharp crystalline peaks in the Ti_3_C_2_‐PSF membrane pattern reflect the successful embedding of MXene flakes into the polymer. The disruption of the crystalline structure is consistent with the random orientation of MXene flakes within the matrix and the loss of long‐range order upon incorporation into the polymer.

The EDX spectrum of the Ti_3_C_2_‐PSF membrane highlights the elemental composition of the material (Figure 4c). The binding energy peaks of Ti appear prominently at approximately 4.508 and 4.923 keV. Other elements detected include carbon (≈0.277 keV) and oxygen (≈0.525 keV), likely originating from the polymeric PSF matrix and potential surface functionalization or oxidation. Sulfur (≈2.307 keV) represents the sulfonate groups within the PSF membrane, providing evidence of the polymer's contribution to the composite. Gold peaks (≈2.120, 9.72, and 11.48 keV) are observed due to the gold coating applied during sample preparation for enhanced conductivity during the EDX analysis.

The deconvoluted XPS spectra for the Ti_3_C_2_‐PSF membrane (Figure 4d) reveal no detectable titanium peaks, with the observed data consisting solely of background noise. Because XPS is surface sensitive (≈1–10 nm) [22], this result indicates that the outermost membrane surface is polymer‐rich and that most Ti_3_C_2_T*_x_* flakes are embedded beneath the air‐facing interface rather than exposed directly at the top surface. This interpretation is consistent with the AFM roughness data and the cross‐sectional TEM/EDX observations. Accordingly, the present data support subsurface incorporation of MXene beneath a polymer‐rich outer surface, but they do not justify attributing the measured contact angle directly to exposed MXene at the air‐facing interface.

The contact angle data presented in Figure 4e provide insight into the apparent wettability of the membranes. The pristine PSF membrane exhibits a relatively high contact angle of around 90°, whereas the Ti_3_C_2_‐PSF composite membrane shows a significantly lower value of around 62°. Considering the absence of Ti signal in XPS and the comparable AFM roughness, we interpret the lower apparent contact angle more conservatively as arising from MXene‐induced changes in near‐surface membrane morphology, higher bulk porosity, and greater water uptake of the composite structure, all of which can influence apparent contact angles measured on porous membranes [23]. This improved apparent wettability is consistent with the higher flux observed for the Ti_3_C_2_‐PSF membrane.

The pristine PSF and Ti_3_C_2_‐PSF membranes exhibit negative zeta potential values, indicating negatively charged surfaces (Figure 4f). However, the Ti_3_C_2_‐PSF membrane demonstrates a slightly more negative charge across a range of pH values, which can be attributed to the negatively charged functional groups (such as ─OH and ─F) on the MXene surface [24]. This increased negative surface charge enhances electrostatic repulsion, reducing the adhesion of negatively charged foulants like humic acid.

### 3.3 Membrane Filtration Performance

To validate the success of this large‐scale manufacturing method, the fabricated membranes were tested in a filtration setup to demonstrate their structural integrity, operability, and performance under realistic conditions.

**FIGURE 5:**
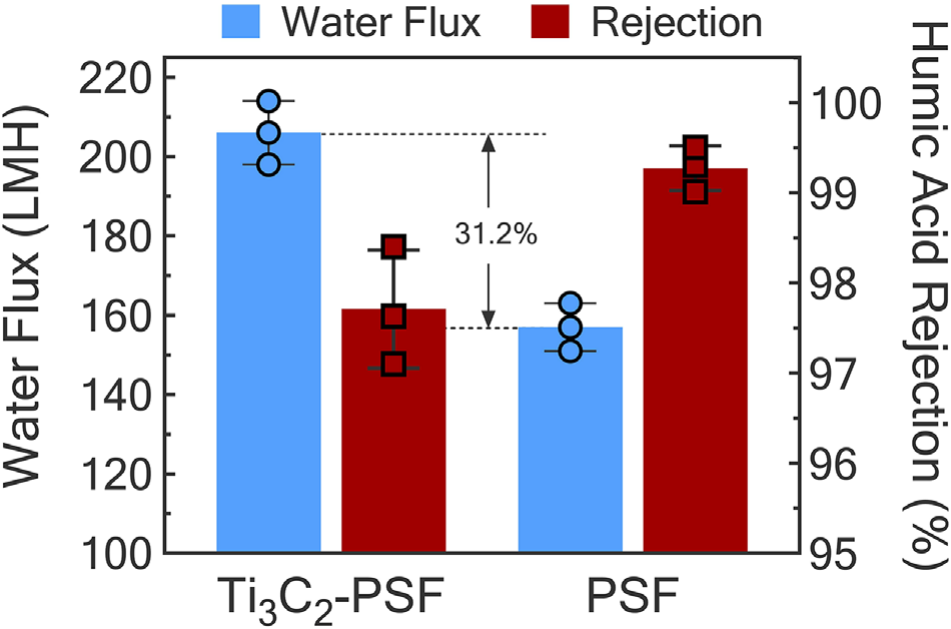
Filtration performance (*n* = 3 independent R2R batches). Mean flux (206 ± 8 LMH for Ti_3_C_2_‐PSF vs 157 ± 6 LMH for PSF; **p < 0.01, Student's t‐test) and humic acid rejection (97.6 ± 0.4% vs 99.3 ± 0.2%; *p < 0.05). Batch‐to‐batch variability remained <5%, indicating reproducible large‐area fabrication.

The filtration performance was assessed using a humic acid solution to verify that the membranes could withstand practical conditions without mechanical failure or performance degradation (Figure 5). The Ti_3_C_2_‐PSF membranes exhibited a flux of 206 LMH, a 31% improvement compared with the 157 LMH recorded for the PSF‐only membranes. This increase in permeability highlights the successful integration of Ti_3_C_2_T*_x_* into the PSF matrix and is consistent with the more open and interconnected internal morphology observed by SEM/TEM. In terms of rejection, the Ti_3_C_2_‐PSF membranes achieved 97.6% humic acid rejection, compared with 99.3% for the PSF membranes. Thus, MXene incorporation produced a clear permeability gain while maintaining high humic acid removal, albeit with a modest decrease in rejection.

### 3.4 Ti_3_C_2_T*_x_* Membrane Fabrication via Nonsolvent Induced Phase Separation (NIPS)

The incorporation of Ti_3_C_2_T*_x_* MXene into the PSF casting solution alters the phase inversion process through coupled thermodynamic–kinetic effects, ultimately governing membrane morphology and transport properties. In conventional NIPS systems, membrane formation is dictated by solvent–nonsolvent exchange, polymer concentration gradients, and the evolution of local thermodynamic instability within the ternary PSF–DMF–water system. These processes are commonly interpreted using a ternary phase diagram framework, where binodal and spinodal boundaries define the onset and mechanism of phase separation [25, 26].

**FIGURE 6:**
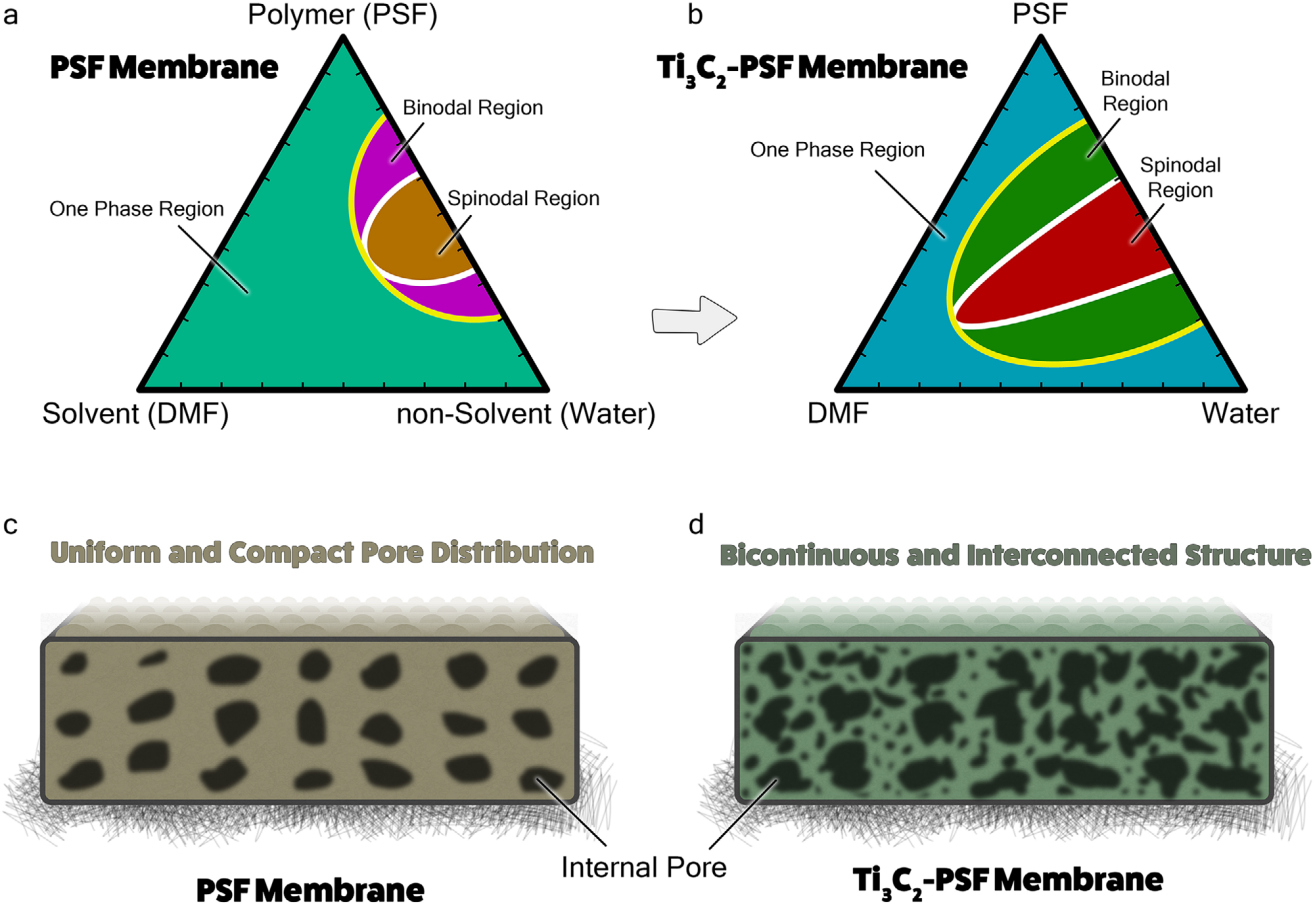
Mechanistic interpretation of Ti_3_C_2_T*_x_*‐induced changes in NIPS membrane formation. (a) Schematic ternary phase diagram of the PSF–DMF–water system showing the conceptual binodal boundary separating single‐phase and two‐phase regions. (b) Schematic comparison illustrating the effective shift in phase separation pathway upon Ti_3_C_2_T*_x_* incorporation. The diagram is a conceptual representation (not to scale, no axis units) and does not represent experimentally measured binodal or spinodal curves. The presence of MXene alters the kinetics of solvent–nonsolvent exchange and local microstructure, leading to earlier entry into the two‐phase region and faster demixing. (c) Schematic structure of the pristine PSF membrane formed under conventional NIPS conditions, characterized by a relatively dense skin layer and limited pore interconnectivity. (d) Schematic structure of the Ti_3_C_2_T*_x_*–PSF membrane, showing a more interconnected porous network resulting from accelerated phase separation and modified formation pathways. MXene flakes are embedded in the subsurface region and influence membrane morphology without being present at the outermost surface.

Figure 6 presents a schematic representation (not to scale) of the ternary phase behavior for PSF systems with and without Ti_3_C_2_T_x_. Importantly, this diagram is used here as a conceptual tool to describe the evolution of the system during phase inversion, rather than a quantitatively measured phase diagram. Within this framework, the addition of Ti_3_C_2_T*_x_* does not directly redefine the intrinsic Flory–Huggins interaction parameter (χ_PSF–DMF_) of the polymer–solvent pair. Instead, MXene acts as a solid, highly hydrophilic, high‐aspect‐ratio additive that modifies the effective phase separation pathway through changes in local composition, transport kinetics, and polymer–filler interactions. Specifically, Ti_3_C_2_T*_x_* influences the NIPS process through three dominant and coupled mechanisms [25, 27, 28]:

- Modification of local solvent–nonsolvent exchange kinetics: The hydrophilic surface terminations of Ti_3_C_2_T*_x_* (─OH, ─O, ─F) enhance water affinity at the nanoscale, promoting accelerated nonsolvent ingress during immersion. This leads to a steeper solvent–nonsolvent exchange gradient, particularly in regions surrounding MXene flakes. As a result, the system is driven more rapidly into the two‐phase region, effectively shifting the precipitation pathway toward earlier demixing, even if the equilibrium binodal itself is not fundamentally altered [25, 27].
- Alteration of dope microstructure and local rheology: The presence of dispersed 2D MXene flakes increases solution viscosity and introduces local heterogeneities in polymer chain packing. These nanoscale perturbations act as microstructural inhomogeneities that influence the onset of phase separation and the spatial evolution of polymer‐rich and polymer‐lean domains. Such effects are well established in mixed‐matrix membrane systems, where particulate additives influence phase inversion through kinetic arrest and domain stabilization, rather than purely thermodynamic shifts [29].
- Coupled thermodynamic–kinetic pathway modification: The combined effect of enhanced water affinity and altered mass transport leads to a more rapid transition through metastable regions of the ternary system, favoring the formation of interconnected, bicontinuous structures. While classical spinodal decomposition cannot be conclusively proven without time‐resolved measurements (e.g., turbidity or light scattering), the observed morphology (Figure 3e,f) is consistent with rapid phase separation pathways that generate highly interconnected porous networks, rather than delayed nucleation‐and‐growth‐dominated structures [25, 26].

This interpretation aligns the morphological observations with the process physics of NIPS, without overextending the thermodynamic claims. Therefore, instead of attributing pore formation strictly to a shift in χ or ΔG_m_, the results indicate that Ti_3_C_2_T*_x_* modifies the effective trajectory through the schematic phase‐inversion map, enabling faster demixing and more efficient pore connectivity.

The structural consequences of this modified phase inversion pathway are evident in Figure 6c,d. The pristine PSF membrane exhibits a relatively dense skin layer with limited pore interconnectivity, characteristic of conventional NIPS under diffusion‐limited conditions. In contrast, the Ti_3_C_2_T*_x_*–PSF membrane displays a more open, interconnected porous architecture, facilitating enhanced water transport. This morphological transition directly correlates with the observed increase in water flux (206 LMH vs 157 LMH), while maintaining high humic acid rejection.

Furthermore, the role of Ti_3_C_2_T*_x_* in this process is primarily subsurface and structural, rather than surface‐dominant. TEM and XPS analyses confirm that MXene flakes are embedded within the membrane matrix (10–500 nm depth), with no detectable presence in the outermost surface layer. Consequently, the improved wettability (contact angle reduction from ≈90° to ≈62°) is more accurately attributed to near‐surface porosity, enhanced water uptake, and increased effective surface area, rather than direct exposure of MXene at the interface. In porous polymeric systems, such changes lead to lower apparent contact angles due to liquid penetration and roughness‐induced wetting amplification, consistent with Wenzel‐type behavior [23, 30].

## 4 Conclusion

This study demonstrates the scalable fabrication of Ti_3_C_2_T*_x_*‐based ultrafiltration membranes using a roll‐to‐roll nonsolvent induced phase separation (R2R‐NIPS) process. The results confirm that Ti_3_C_2_T*_x_* incorporation significantly influences the membrane formation pathway, leading to measurable changes in structure, wettability, and filtration performance.

Rather than directly altering equilibrium thermodynamic parameters, Ti_3_C_2_T*_x_* modifies the effective phase inversion process through coupled kinetic and microstructural effects, including enhanced solvent–nonsolvent exchange, local viscosity changes, and nanoscale heterogeneity in the casting solution. These effects collectively promote a more rapid demixing pathway and the formation of a highly interconnected porous structure, which is consistent with the observed morphological features. Comprehensive characterization confirms that MXene flakes are embedded within the membrane matrix, predominantly in subsurface regions, while the outermost surface remains polymer‐dominated. The improved hydrophilicity of the composite membrane is therefore attributed to near‐surface structural changes, increased porosity, and enhanced water uptake, rather than direct surface exposure of MXene. As a result, the Ti_3_C_2_T*_x_*–PSF membranes exhibit a substantial increase in water permeability (≈30% higher flux) while maintaining high rejection performance. Importantly, the successful implementation of the R2R‐NIPS process demonstrates the feasibility of translating MXene‐based membranes from laboratory‐scale fabrication to continuous, large‐area production.

This work establishes a scalable processing framework for MXene‐integrated membranes and provides mechanistic insight into how 2D nanomaterials influence phase inversion. These findings offer a practical pathway toward the development of high‐performance, industrially relevant membrane systems for water treatment applications.

## Conflicts of Interest

The author declares no conflicts of interest.

## Supporting information




**Supporting File**: smtd70676‐sup‐0001‐SuppMat.docx.

## Data Availability

The data that support the findings of this study are available from the corresponding author upon reasonable request.
