# Supplementary material for: Large‐Scale MXene Membrane Fabrication via Nonsolvent Phase Separation
Source: Small Methods. 2026 May 1;10(14):e02288. doi: 10.1002/smtd.202502288 (PMC13397248; doi:10.1002/smtd.202502288)
Supplement: Supplementary file 1 — Supporting File: smtd70676‐sup‐0001‐SuppMat.docx. [file SMTD-10-e02288-s001.docx]

Supporting Information (SI):

Large-Scale MXene Membrane Fabrication via Nonsolvent Phase Separation

Mostafa Dadashi Firouzjaei ^[a,b]^*, Zahra Zandi ^[b]^, Hesam Jafarian ^[a]^, Anupma Thakur ^[c]^, Sanam Etemadi Maleki ^[a]^, Ahmad Rahimpour ^[c]^, Ahmad Arabi Shamsabadi ^[d]^, Mohtada Sadrzadeh ^[c]^*, Babak Anasori ^[c,e]^*, and Mark Elliott ^[a]^*

^[a]^ Department of Civil, Construction, and Environmental Engineering, University of Alabama, Tuscaloosa, AL, 35487, USA

^[b]^ Department of Mechanical Engineering, 10-241 Donadeo Innovation Center for Engineering, Advanced Water Research Lab (AWRL), University of Alberta, Edmonton, AB, T6G 1H9, Canada

^[c]^ School of Materials Engineering, Purdue University, West Lafayette, IN 47907, USA.

^[d]^ Department of Chemistry, University of Pennsylvania, Philadelphia, PA 19104, USA.

^[e]^ School of Mechanical Engineering, Purdue University, West Lafayette, IN 47907, USA.

***Corresponding Authors**:

Mostafa Dadashi Firouzjaei ([mdfirouzjaei@ua.edu](mailto:mdfirouzjaei@ua.edu))

Mohtada Sadrzadeh ([sadrzade@ualberta.ca](mailto:sadrzade@ualberta.ca))

Babak Anasori (banasori@purdue.edu)

Mark Elliott (melliott@eng.ua.edu)

# Membrane Fabrication Method and Variables

The fabrication of large-scale Ti_3_C_2_T*_x_* MXene membranes was achieved using a R2R-NIPS casting technique developed and customized in-house. This section is presented as practical processing guidance rather than as a formal quantitative optimization study. The parameter values reported below were selected after iterative trials on the basis of processability, coating stability, defect suppression, and reproducible membrane recovery during continuous casting.

## Roll-to-Roll Casting Machine and Polymer Solution Preparation

The R2R-NIPS casting machine was designed to enable continuous fabrication of membranes with precise control over thickness, casting speed, and other critical parameters. Polyester was used as the substrate for membrane formation due to its mechanical robustness and compatibility with the polymer solution. The casting head of the machine was adjustable, allowing fine control over the wet film thickness, which is essential for achieving consistent membrane properties across large areas. Two types of polymer solutions were prepared for this study: (a) PSF without MXene and (b) Ti₃C₂-PSF mixed solution.

## Ti_3_C_2_T*_x_* MXene Membrane Casting Process

The solution was poured directly under the adjustable casting knife, which spread the polymer solution evenly onto the polyester substrate as it moved through the casting system. This manual application step ensured that sufficient material was supplied for continuous casting while preventing overflow or inconsistencies at the casting head. The deposited film thickness was precisely controlled using the casting knife, which was calibrated to the desired gap height (set at 200 μm in this work). Following deposition, the polymer-coated substrate entered a coagulation bath containing 30 liters of deionized (DI) water maintained at a constant temperature of 21 °C. In this work, the default fabrication conditions were a 200 μm wet gap, 2.5 m min⁻¹ line speed, and 2.0 L min⁻¹ bath circulation. These conditions were chosen because they provided stable coating and reproducible membrane formation under continuous operation.

### Effect of Casting Speed

Due to the high aspect ratio and hydrophilic nature of Ti_3_C_2_T*_x_* MXenes, the dispersion behavior of the MXene flakes in the polymer matrix during casting is particularly sensitive to the casting speed at which the polymer solution is spread. At higher casting speeds, the shear forces exerted on the polymer solution increase, which can enhance the alignment of MXene flakes within the membrane. This alignment is advantageous for applications requiring anisotropic properties, such as enhanced conductivity or selective separation, as the MXene layers tend to orient parallel to the membrane surface. However, excessively high speeds can lead to incomplete wetting of the substrate and potential discontinuities in the polymer film, resulting in defects or non-uniform membrane thickness. Such issues can compromise the separation performance and mechanical integrity of the final membrane.

Conversely, slower casting speeds allow for a more controlled and uniform deposition of the polymer solution, promoting a more random distribution of MXene flakes within the matrix. While this can enhance isotropic properties, such as overall mechanical strength and bulk hydrophilicity, slower speeds also increase the risk of solvent evaporation prior to the coagulation bath. Premature evaporation of DMF alters the phase inversion kinetics, potentially leading to a denser, less porous membrane structure, which can negatively affect water flux.

For this study, a casting speed of 2.5 m/min was selected from iterative line trials because it provided stable coating and uniform film formation while minimizing visible defects. We therefore present this value as a practical processability setting rather than as a uniquely optimized condition. Adjustments to the casting speed were also made based on the viscosity of the polymer solution, as the addition of MXenes increased the solution's resistance to flow, requiring careful calibration to avoid defects in the final membrane.

### Effect of Casting Thickness

A thicker casting layer increases the availability of the polymer and MXene material, which can enhance the membrane's mechanical strength and durability. In the case of Ti_3_C_2_T*_x_*, the increased casting thickness allows for more uniform dispersion of the flakes within the matrix, reducing the likelihood of aggregation and ensuring consistent functional properties across the membrane. However, excessively thick layers can delay solvent exchange during the phase inversion process, leading to irregular pore formation and reduced porosity. This can compromise water flux and reduce the efficiency of separation.

On the other hand, thinner casting layers promote faster solvent diffusion during phase inversion, which can result in higher porosity and improved water flux. However, this also poses challenges specific to MXene-polymer systems. Thinner layers increase the shear forces during deposition, which can exacerbate MXene misalignment or result in uneven distribution of the flakes, particularly if the solution's viscosity is high. Additionally, thin films are more prone to mechanical fragility and cracking, especially during post-fabrication handling and testing.

In this study, a wet thickness of 200 μm was selected from iterative casting trials because it consistently produced continuous, defect-minimized membranes with adequate mechanical integrity. We therefore describe this value as a practical operating condition rather than a quantitatively optimized global optimum. The casting knife calibration was routinely checked to maintain this thickness and ensure reproducibility across the fabricated membranes.

### Effect of Substrate Tension

Maintaining optimal tension in the polyester substrate is essential to ensure a smooth and uniform casting surface. Insufficient tension can lead to wrinkling or sagging of the substrate, causing irregularities in the deposited film and uneven distribution of MXene flakes within the membrane. Such defects are particularly detrimental for Ti_3_C_2_T*_x_*-based membranes, as nonuniformity in MXene dispersion can result in inconsistent separation performance and localized mechanical weaknesses. Excessive substrate tension, on the other hand, can cause excessive stretching or deformation of the polyester substrate. This introduces shear stress in the polymer solution during deposition, which can disrupt the alignment of MXene flakes or lead to microscale defects such as holes or thinning in the membrane structure. Furthermore, high tension may compromise the adhesion between the membrane and the substrate, increasing the risk of delamination during phase inversion or post-fabrication handling.

### Effect of Bath Circulation

Adequate circulation within the coagulation bath is essential to maintain uniform solvent concentration and temperature throughout the bath. Poor circulation can result in localized variations in the solvent exchange rate, leading to uneven pore formation or defects in the membrane structure. For Ti_3_C_2_T*_x_*-incorporated membranes, such inconsistencies can disrupt the dispersion and orientation of MXene flakes within the polymer matrix, affecting both the final membrane's separation performance and mechanical properties. Overly vigorous circulation, however, can introduce turbulence that disrupts the delicate phase inversion process. This may lead to surface roughness or irregularities in the membrane structure, which are particularly problematic for MXene-based membranes, where uniformity in pore size and flake distribution is critical for functional performance. In this study, the coagulation bath had a controlled circulation system (flow rate of 2.0 L/min) to maintain consistent DI water flow and temperature. The bath was maintained at 21 °C, ensuring stable phase inversion kinetics without inducing thermal stress on the polymer-MXene matrices.

### Effect of Uniform MXene Dispersion

The high aspect ratio, strong interlayer Van der Waals interactions, and hydrophilic nature of Ti_3_C_2_T*_x_* make them prone to aggregation in the polymer matrix, which can lead to defects, uneven mechanical properties, and inconsistent separation performance in the final membrane.

Incorporating MXenes into the polymer solution required careful optimization of the mixing process. In this study, uniform dispersion was achieved by gradually adding MXene powder into the DMF-based polymer solutions under continuous stirring. Moderate stirring speeds were employed for a sufficient duration to promote uniform dispersion without introducing excessive shear forces. The solution was stirred for 6 hours to ensure the homogeneous distribution of MXenes throughout the matrix. The viscosity of the polymer solution was another key factor influencing MXene dispersion. The presence of MXenes increases the viscosity of the solution due to their high surface area and strong interactions with the polymer matrix. This necessitated adjustments to the stirring speed and duration to prevent localized agglomeration of MXene flakes while maintaining the solution’s flowability for casting. After mixing, vacuum degassing further ensured that air bubbles, which could trap MXene aggregates or lead to defects in the membrane, were removed.

The R2R-NIPS process timeline—from dope casting to complete membrane solidification in the coagulation bath—was shorter than 20 min for a given run, limiting the time available for aggregation or oxidation during fabrication. The coating step itself (solution spreading to bath immersion at 2.5 m/min) lasted only seconds in a DMF-rich environment. Within this processing window, no obvious aggregation-related defects were observed by SEM/TEM. We emphasize, however, that this practical observation does not substitute for a dedicated oxidation-kinetics study.

## Post-Fabrication Handling

Following phase inversion, the membranes were thoroughly rinsed with DI water to remove residual solvents, such as DMF, and any unreacted components. The rinsing process was conducted in a clean environment to prevent contamination, which could compromise the membrane’s separation performance or surface properties. Multiple rinsing cycles ensured the complete removal of impurities, a particularly critical step for MXene-containing membranes, where surface functionality plays a significant role in performance. The rinsed membranes were then stored in DI water at room temperature to maintain hydration and prevent drying. Drying of the membranes was avoided, as it could lead to cracking or deformation, especially in the Ti_3_C_2_T*_x_* layers, which are sensitive to changes in moisture content. To further ensure membrane stability, storage containers were sealed to minimize exposure to air and environmental fluctuations.

**Table S1.** Representative scalable or continuous-fabrication MXene-based products reported in the literature.

| Product | Method | Application Focus | MXene | Year | Ref |
| --- | --- | --- | --- | --- | --- |
| Membrane^1^ | Slot-die coating | Nanofiltration membranes, adsorbents, sensors, Electromagnetic Interference (EMI) shielding, and energy devices | Ti_3_C_2_T*_x_* | 2021 | ^[1]^ |
| Fiber | Continuous wet spinning | Electrical wires for switching on an LED light and transmitting electrical signals to earphones | Ti_3_C_2_T*_x_* | 2020 | ^[2]^ |
| Fiber | Continuous wet spinning + thermal drawing | Wearable textiles, EMI shielding, and electrothermal management | Ti_3_C_2_T*_x_* | 2022 | ^[3]^ |
| Fiber^2^ | Blade coating + sol-gel conversion | EMI shielding, thermal management, and flexible electronics | Ti_3_C_2_T*_x_* | 2022 | ^[4]^ |
| Aerogel | Metal ion-induced assembly + freeze drying on shrink film substrate | EMI shielding, supercapacitors, and Capacitive Deionization (CDI) | Ti_3_C_2_T*_x_* | 2021 | ^[5]^ |
| Aerogel^3^ | Hydrothermal assembly + natural drying + Joule annealing | Pressure sensors and wearable electronics | Ti_3_C_2_T*_x_* | 2024 | ^[6]^ |
| Electrode array | Selective wetting and UV/ozone treatment combined with solution processing (dip and dry) | Electrodes (source, drain, gate) in p-type and n-type Organic Field-Effect Transistors (OFETs) | Ti_3_C_2_T*_x_* | 2019 | ^[7]^ |
| Heterostructured paper^4^ | Spray-assisted layer-by-layer assembly | Energy storage, flexible electronics, and EMI shielding | Ti_3_C_2_T*_x_* | 2019 | ^[8]^ |
| Film | Blade coating | EMI shielding and current collectors for  batteries and supercapacitors | Ti_3_C_2_T*_x_* | 2020 | ^[9]^ |
| Film^5^ | Blade coating + drying + GO reduction | Flexible electronics and EMI | Ti_3_C_2_T*_x_* | 2021 | ^[10]^ |
| Cellulose yarn^6^ | Two-step dip-coating | Wearable electronics, sensors, and EMI shielding | Ti_3_C_2_T*_x_* | 2019 | ^[11]^ |

1. MXene coating on a substrate of PES
2. MXene/Aramid nanofiber
3. MXene/rGO aerogel
4. MXene/rGO heterostructures
5. S, N-codoped MXene/rGO composite film
6. MXene-coated cellulose yarns

**References**

[1] J. H. Kim, G. S. Park, Y.-J. Kim, E. Choi, J. Kang, O. Kwon, S. J. Kim, J. H. Cho, D. W. Kim, *ACS Nano* **2021**, 15, 8860.

[2] W. Eom, H. Shin, R. B. Ambade, S. H. Lee, K. H. Lee, D. J. Kang, T. H. Han, *Nature Communications* **2020**, 11, 2825.

[3] T. Zhou, Y. Yu, B. He, Z. Wang, T. Xiong, Z. Wang, Y. Liu, J. Xin, M. Qi, H. Zhang, X. Zhou, L. Gao, Q. Cheng, L. Wei, *Nature Communications* **2022**, 13, 4564.

[4] J. Wang, X. Ma, J. Zhou, F. Du, C. Teng, *ACS Nano* **2022**, 16, 6700.

[5] M. Ding, S. Li, L. Guo, L. Jing, S.-P. Gao, H. Yang, J. M. Little, T. U. Dissanayake, K. Li, J. Yang, Y.-X. Guo, H. Y. Yang, T. J. Woehl, P.-Y. Chen, *Advanced Energy Materials* **2021**, 11, 2101494.

[6] W. Zhu, Y. Zhuang, J. Weng, Q. Huang, G. Lai, L. Li, M. Chen, K. Xia, Z. Lu, M. Wu, Z. Zou, *Advanced Materials* **2024**, 36, 2407138.

[7] B. Lyu, M. Kim, H. Jing, J. Kang, C. Qian, S. Lee, J. H. Cho, *ACS Nano* **2019**, 13, 11392.

[8] M.-Q. Zhao, N. Trainor, C. E. Ren, M. Torelli, B. Anasori, Y. Gogotsi, *Advanced Materials Technologies* **2019**, 4, 1800639.

[9] J. Zhang, N. Kong, S. Uzun, A. Levitt, S. Seyedin, P. A. Lynch, S. Qin, M. Han, W. Yang, J. Liu, X. Wang, Y. Gogotsi, J. M. Razal, *Advanced Materials* **2020**, 32, 2001093.

[10] L. Liao, D. Jiang, K. Zheng, M. Zhang, J. Liu, *Advanced Functional Materials* **2021**, 31, 2103960.

[11] S. Uzun, S. Seyedin, A. L. Stoltzfus, A. S. Levitt, M. Alhabeb, M. Anayee, C. J. Strobel, J. M. Razal, G. Dion, Y. Gogotsi, *Advanced Functional Materials* **2019**, 29, 1905015.
